# Broad-Spectrum Drugs Against Viral Agents

**DOI:** 10.3390/ijms9091561

**Published:** 2008-09-01

**Authors:** Mary E. Christopher, Jonathan P. Wong

**Affiliations:** Defence Research & Development Canada – Suffield, Box 4000, Station Main, Medicine Hat, AB, Canada T1A 8K6. E-Mail: Jonathan.Wong@drdc-rddc.gc.ca (J. W.)

**Keywords:** CpG, cytokine, influenza, poly (ICLC), TLR

## Abstract

Development of antivirals has focused primarily on vaccines and on treatments for specific viral agents. Although effective, these approaches may be limited in situations where the etiologic agent is unknown or when the target virus has undergone mutation, recombination or reassortment. Augmentation of the innate immune response may be an effective alternative for disease amelioration. Nonspecific, broad-spectrum immune responses can be induced by double-stranded (ds)RNAs such as poly (ICLC), or oligonucleotides (ODNs) containing unmethylated deocycytidyl-deoxyguanosinyl (CpG) motifs. These may offer protection against various bacterial and viral pathogens regardless of their genetic makeup, zoonotic origin or drug resistance.

## 1. Introduction

Influenza viruses are a major concern for public health officials due to their potential to cause pandemics. In recent years, influenza viruses A/H2, A/H5, A/H7 and A/H9 [1] have resulted in human illness as the result of poultry-to-human transmission. At present, these viruses are not readily spread from human-to-human, but a mutated and/or reassorted virus with efficient human-to-human transmission could trigger an influenza pandemic. Should the avian H5N1 influenza virus currently circulating in Asia assume the role of a pandemic agent, formidable technical difficulties relating to the properties of the virus itself, vaccine production, distribution and administration would ensure that vaccines would only become available after a significant lead time, with a low likelihood that a vaccine would be available during the first wave of the pandemic [2, 3]. The use of existing antivirals, primarily oseltamivir (Tamiflu) could be critical in the initial control of a pandemic [3–5], although there have been reports of oseltamivir resistance in influenza H5N1 infected patients [3, 6, 7]. Drug resistance in influenza strains is increasing worldwide as evidenced by a survey of influenza H3N2, H1N1 and H1N2 field isolates. A significant increase in resistance to Amantadine and Rimantadine from 0.4% in 1994–1995 to 12.3% in 2003–2004 was observed with 61% of samples obtained in Asia since 2003 being drug resistant [8].

Mucosal surfaces serve as the entry sites for the majority of infectious pathogens, including influenza viruses, and provide the first line of defence against infection [9]. During the early stages of infection, the immune response is non-antigen specific, involving natural killer (NK) and natural killer T cells (NKT cells) which, through the activation of antigen presenting cells (APCs), indirectly respond to danger signals derived from invading pathogens. Macrophages and dendritic cells (DCs) express numerous Toll-like receptors (TLRs) [10] and respond to microbial pathogens by producing type I interferons (IFN) and cytokines. There are ten different TLRs, each binding specific classes of compounds. Unmethylated CpG dinucleotides present in bacterial DNA are recognized by TLR9 [11–13], single-stranded (ss)RNA viruses, such as vesicular stomatitis virus and influenza virus are recognized by TLR7 [14], and dsRNA longer than 30 base pairs, produced during the viral replicative cycle, is recognized by TLR3 [11, 13, 15, 16].

In situations where a vaccine is unavailable and/or drug resistance is prevalent, it would be advantageous to stimulate the immune system to non-specifically respond to viral threats. Poly (ICLC), a dsRNA, and CpG ODNs, molecular mimics for TLR3 and TLR9, respectively, have been reported to non-specifically stimulate the innate immune system and to provide protection against various bacterial and viral pathogens, including influenza. In addressing whether these agents could potentially be used as therapeutic agents or vaccine adjuvants should an influenza H5N1 pandemic arise, we need to understand the mechanism of action of poly (ICLC) and CpG ODNs in relation to the pathogenic effects of influenza H5N1.

## 2. TLR3 agonists: dsRNA

Cells are armed with various latent mechanisms that are able to sense viral components and initiate intracellular signal transduction to respond rapidly to viral infections. Alveolar and bronchial epithelial cells, the primary target and principal host cells for influenza viruses, play a key role in the initiation of innate and adaptive immune responses to influenza virus [17, 18]. Double-stranded RNA produced during viral replication is recognized by an intracellular protein, TLR3, present in T, NK [15, 19, 20], mast [21] and epithelial cells [17]. In humans, TLR3 mRNA is constitutively expressed in alveolar and bronchial epithelial cells, placenta, pancreas, liver, spleen, heart, brain and intestine, and is up-regulated by influenza virus [17, 22]. Recognition by TLR3 results in enhanced production of IFN-α, -β, -γ, and -λ, IL-1β, -6, -8, -12, -15 and -18, IP-10, GM-CSF, regulated on activation, normal T cells expressed and secreted (RANTES), LARC, MIP-1α, GRO-α, ENA-78, ICAM-1 (which recruits inflammatory cells) and nitric oxide synthase [15, 17, 21, 23–29], as well as stimulation of specific components of the cellular and humoral immune systems, including NK cell activation [30, 31] and memory CD8+ T cell proliferation [15]. Additionally, dsRNA-activated enzymes such as RNA helicases (RIG-1 and MDA-5), protein kinases (PKR), 2′-5′-oligoadenylate synthetase and RNA-specific adenosine deaminase which can directly inhibit viral replication are also induced. Viruses evade these enzymes either by expressing dsRNA-binding proteins or by inhibiting dsRNA-induced pathways [16].

Synthetic dsRNAs including polyriboinosinic-polyribocytidylic acid (poly (IC)) alone or stabilized with poly-L-lysine carboxymethyl cellulose (poly (ICLC)), polyadenosinic-polyuridylic acid (poly (AU)) and Ampligen (polyI:polyC(12)U) have been used as TLR3 molecular mimics [24, 30, 32, 33] and TLR3 expression is up-regulated by these molecules [17]. It has recently been demonstrated that TLR3 also binds polyriboinosinic acid, a ssRNA [34]. A comparative study in mice using poly (ICLC), poly (IC) and poly (AU) determined that IFN induction was highest with poly (ICLC) and lowest with poly (AU), with splenocyte cytotoxicity being significantly higher with poly (ICLC) than with poly (AU) [35]. Following intravenous (i.v.) infusion of poly (ICLC), substantial increases in total IFN levels were observed 8 to 16 h later in humans and Rhesus monkeys, with males of both species having consistently and significantly higher IFN responses [36, 37]. The response to poly (IC) in the human tracheobronchial epithelial cell line BEAS-2B was approximately 20 h earlier than in the same cells infected with influenza A/Scotland/20/74 (H3N2), possibly reflecting the time required to generate dsRNA within viral-infected cells [17]. Interestingly. although TLR3 is critical for IFN-γ production from CD8+ T cells, TLR7 has been implicated in the IFN-α response to poly (AU) in murine plasmacytoid DCs (pDCs) [38].

Poly (ICLC) has been shown to be effective in protecting rodents against influenza virus [32], Rift Valley fever virus [25], rabies virus [39], Punta Toro virus [40] and western equine encephalitis virus [41]. It also protects the marine crustacean *Litopenaeus vannamei* from infections of white spot syndrome virus and Taura syndrome virus [42]. In primates, poly (ICLC) is effective against yellow fever virus [43], Venezuelan equine encephalomyelitis virus (VEEV) [44] and rabies virus [39]. Poly(IC) has also been shown, through its effects on NK cells, to eliminate the histological lesions of graft-versus-host disease in mice [45]. Ampligen, a mismatched double-stranded RNA currently under development by Hemispherx Biopharma in the U.S.A., acts by inducing IFN production (immunomodulator) and by activating an intracellular enzyme (RNase-L) against viral RNA transcripts (antiviral). Ampligen is indicated for the treatment of chronic fatigue syndrome and acquired immunodeficiency deficiency syndrome (AIDS) as part of the combination therapy. In May 2004 Hemispherx announced that it had filed an expanded U.S.A. patent application covering its use for the potential treatment and prevention of severe acute respiratory syndrome (SARS) and other emerging viruses [46]. Ampligen has been determined to be safe in phase III human trials.

### 2.1. Poly (ICLC) as prophylaxis

**Figure 1.**
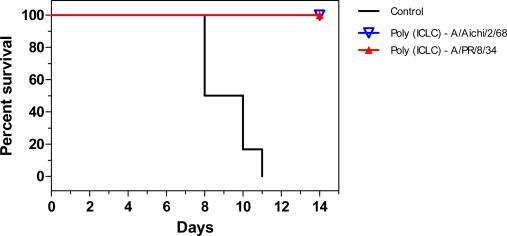
Efficacy of poly (ICLC) prophylaxis against influenza viral challenge. Mice were given 20 μg (1 mg/kg body weight) of poly (ICLC) intranasally 48 and 8 h prior to challenge with a lethal dose of influenza A/PR/8/34 or A/Aichi/2/68. Liposomes were composed of PC/CH/SA in a 7:2:1 molar ratio. Mice (n=6) were monitored daily for appearance, weight and survival. Graph shows the percentage of mice surviving 14 days after challenge. The experiment was done twice. Adapted from [47].

In mice, two doses of 1 mg/kg/dose poly (ICLC) given intranasally (i.n.) provided complete protection against influenza A/PR/8/34 (H1N1) or A/Aichi/2/68 (H3N2) viral challenge (Figure 1), whereas those pre-treated with a single dose had a slightly lower survival rate of 80% [32, 47]. Comparative studies demonstrated that two i.n. doses (1 mg/kg/dose) of poly (ICLC) was more effective than two i.n. doses (100,000 U/kg/dose) of either recombinant mouse IFN-α or -γ in protection of mice against influenza A/PR/8/34 infection, with survival rates of 100% and 50%, respectively [32]. Mice treated with two doses of poly (ICLC), 48 h apart, up to 12 d prior to viral challenge were completely protected from infection, whereas survival rates decreased to 80, 40 and 0%, when pre-treatment was given 14, 16 or 20 d prior to virus challenge, respectively [32]. This increased survival, together with the observation that NK cell activity remained elevated for 9 and 6 d post-poly (ICLC) treatment in liver and blood/spleen, respectively [31], suggests that poly (ICLC) may provide short-term prophylaxis against influenza in an outbreak situation. Poly (AU) also increased NK cell activity in the liver but approximately a 10-fold higher dose was required than for poly (ICLC) [31].

Poly (ICLC) was able to provide protection to mice infected with Rift Valley fever virus provided that the mice were given two to three intraperitoneal (i.p.) doses with one dose being administered prior to infection [25, 48, 49]. A single i.p. injection of Ampligen administered either 24 h or 4–6 h before infection with Banzi virus provided significant improvements in survival. In comparison, Ampligen administered prior to infection with Semliki Forest virus was able to significantly improve mortality when given 4–6 h, but not 24 h, prior to infection [50]. Poly ICLC treatment decreased the number of VEEV-infected monkeys that become detectably viremic and delayed the onset of viremia in the remaining monkeys [44]. When mice were dosed i.p. with 10 mg/kg Ampligen 4 h prior to SARS-CoV exposure, viral titres in the lungs were below detectable limits [51].

Poly (ICLC) administered following influenza viral infection was less effective than when administered prophylactically. Mice treated with two i.v. doses (1 mg/kg/dose) of poly (ICLC) 8 and 48 h post-infection (p.i.) showed a small increase in survival (40%) relative to untreated control mice and post-exposure treatment with a single dose was found to be almost completely ineffective [32]. Efficacy of post-exposure poly (ICLC) treatment has primarily been observed when given in conjunction with pre-exposure treatment(s). Efficacy has been reported for mice infected with Punta Toro virus [40] and West Nile virus when treatments were started 1 d prior to infection, continuing every 48 h until 5 d post-infection. When treatment was delayed until 4–6 h before viral challenge, efficacy was greatly reduced [52]. For treatment of Rift Valley fever at least four doses were required when treatment was started 24 h p.i. [53].

### 2.2. Poly (ICLC) as an adjuvant

Poly (IC) has also been used as an adjuvant with split-product influenza vaccines. Co-administration of poly (IC) with the primary and booster doses of vaccine followed by challenge with influenza A/PR/8/34 resulted in cross-protection when administered with various H1N1 virus vaccines (A/PR/8, A/Beijing, A/Yamagata), partial protection with heterologous influenza A vaccines (A/Guizhou – H3N2) and no protection with influenza B vaccines (B/Ibaraki, B/Yamagata, B/Aichi) [54]. T-cell activation and increased IFN-γ production was observed only in mice immunized with homologous antigens [55]. Treatment with vaccine plus poly (IC) rapidly up-regulated TLR3 expression in the nasal-associated lymphoid tissue, up-regulated IL-4 and IL-12p40, and induced IFN-α, -β and -γ suggesting that route of administration of vaccine plus poly (IC) was important [55]. A trivalent inactivated influenza vaccine co-administered with Ampligen provided cross-protection against various strains of H5N1 influenza virus (A/HongKong/483/97, A/Vietnam/1194/04 and A/Indonesia/6/05) when administered i.n. but not when given subcutaneously (s.c.), confirming the importance of the route of administration [56, 57].

Poly (ICLC) adjuvant activity has been shown when co-administered with retinoic acid [58], VEEV vaccine [59, 60] and the antimalarial drug chloroquine [61]. Synergism of poly (ICLC) with various anti-HIV compounds including cytokines (rIFN-α A, rIFN-β Ser 17, and rIFN-γ), reverse transcriptase inhibitors (azidothymidine and phosphonoformate (Foscarnet)), mRNA capping inhibitors (ribavirin), lipophile (amphotericin B) and glucosidase inhibitor (castanospermine) was observed [62]. Similarly, priming with either IFN-α/β or poly (IC) completely blocked or transiently reduced West Nile virus replication in macrophages from resistant mice or susceptible mice, respectively. Combined pre-treatment with IFN-α/β and poly (IC) elicited strong antiviral responses that completely prevented flavivirus replication in macrophages from susceptible mice [63]. Anti-Semliki Forest virus hyperimmune serum or poly (ICLC) given i.p. were not protective when used alone following an intracranial Semliki Forest virus infection, but when given together survival rate increased to 50% and viremia and viral load in the brain became undetectable [64].

A phase I study of poly (ICLC), in combination with IL-2, in patients with a variety of cancers showed moderate toxicity of poly (ICLC) at all doses tested. No increases in peripheral blood NK cell activity was observed after treatment with poly (ICLC) alone but high doses of poly (ICLC) (≥ 0.3 mg/m^2^) in combination with IL-2 resulted in NK cell activity greater than that seen using the same dose of IL-2 in combination with lower poly (ICLC) doses [65].

### 2.3. Poly (ICLC) safety

The potential of poly (ICLC) as an anti-influenza agent is, however, limited by its intrinsic toxicity. Toxicity of poly (ICLC) is affected by the route of administration with s.c. administration being well tolerated in rabbits [66] and mice (unpublished observations) and intratracheal administration being well tolerated in mice [67]. Intranasal, intramuscular (i.m.), i.v. or i.p. administration results in variable levels of toxicity depending on the animal species being used. In clinical trials, patients receiving multiple therapeutic doses of poly (ICLC) i.v. or i.m. exhibited serious toxic reactions including hypotension, fever, anemia, leukopenia, thrombocytopenia, nausea, injection site inflammation and, in multiple sclerosis patients, neurological dysfunction [54, 68–77]. Intra-articular administration induced arthritis, mediated by IL-1 receptor (IL-1R) signalling, as early as 3 d post-administration [78].

Attempt to improve safety and efficacy of poly (ICLC) have focused on optimization of dosage and treatment regimes, encapsulation within liposomes, modification of poly (ICLC), and co-administration of agents that mitigate cytokine-mediated adverse reactions. In patients with advanced cancer, poly (ICLC) administered on an alternate-day schedule with gradual dose escalation was tolerated the best with the maximum tolerated dose varying over a several hundredfold dose range [69].

In mice, poly (ICLC) administration results in loss of up to 10% of the total body weight and hypothermia of up to 2°C [44]. To mitigate the toxicity of poly (ICLC) without adversely affecting its biological activities, poly (ICLC) has been encapsulated within cationic liposomes composed of phosphatidylcholine, cholesterol (CH) and stearylamine. Intranasal administration of 20 μg free or liposome-encapsulated poly (ICLC) at –3 and –1 d, followed by challenge with a lethal dose of influenza A/PR/8/34 at 0 d resulted in no demonstrable reduction in weight loss following administration or influenza challenge, but the magnitude and duration of body temperature reduction was reduced [47].

Liposome-encapsulation completely mitigated the toxicity (as determined by absence of weight loss and changes in body temperature) seen with free poly (ICLC) when administered i.v. [47]. Mice that received pre-treatment with liposome-encapsulated poly (ICLC) 21 d prior to virus challenge were fully protected whereas mice given free poly (ICLC) were completely protected if treatment was within 12 d of infection [47]. Liposomes have been shown to accumulate at sites of infection [79], possibly concentrating the encapsulated drug at the diseased site and thereby minimizing the exposures of healthy organs and tissues to the drugs. Together, these results suggest that liposome encapsulation results in a gradual and sustained release of poly (ICLC), thereby avoiding rapid systemic elevation of drug levels observed with some routes of administration. It is unclear whether the significant reductions in the toxicity of poly (ICLC) provided by liposomes will result in corresponding decreases in clinical side effects seen in human patients. Such attenuation of the toxic side effects in patients may result in increase drug tolerance and improve clinical outcomes.

Mitigation of cytokine-mediated adverse reactions has been attempted using IL-1R signalling pathway agonists and hydrocortisone treatment. The IL-1R signalling pathway, stimulated by poly (ICLC), is implicated in increased plasma IL-6 concentrations. Male rats given IL-1 receptor agonist (IL-1ra) prior to poly (IC) administration had elevated plasma TNF-α, but not IL-6, concentrations with a concomitant reduction in fever [80]. Hydrocortisone treatment prior to or following i.v. poly (ICLC) administration reduced both the hypotensive responses and interferon induction in rabbits [66].

Rabbits mimic the human febrile and hypotensive response to poly (ICLC) [66] and thus would be useful experimental models to evaluate mechanisms / approaches to reduce poly (ICLC)-mediated toxicity. A lower molecular weight (4S) poly (ICLC) was able to generate high titers of IFN and ameliorated hypotensive responses in rabbits, however, it also induced high fevers [66]. Selective thiolation of the poly (C) strand at the five position of the cytosine base, generates a partially thiolated poly (C) (MPC) which, after annealing with a complentary unmodified poly (I), forms the thiolated dsRNA, pI:MPC. Optimal antiviral and antiproliferative activities were obtained when thiolation was at 7.4% [81]. These results suggest that further study into modification of poly (ICLC) may be advantageous for the elimination / reduction of toxic side effects.

## 3. TLR9 agonists: CpG ODNs

CpG dinucleotides are under-represented in vertebrate DNA (1 in 64 base pairs) and are generally methylated on the cytosine whereas bacterial DNA contains unmethylated CpG dinucleotides at the expected frequency of 1 in 16 base pairs [11]. CpG dinucleotide-containing DNA or CpG-containing oligonucleotides (CpG ODNs) are rapidly internalized by immune cells through the endocytosis / phagocytosis pathway where they interact with the constitutively expressed receptor, TLR9, present in endocytic vesicles, triggering swelling and acidification of the vesicle and generating reactive oxygen species [10, 12, 87]. Activation of the CpG ODN/TLR9 signalling pathway culminates in the activation of several transcription factors [10, 88, 89] which directly up-regulate production of pro-inflammatory cytokines (IL-1, -6, -10, -12 -18, GM-CSF, TNF-α, IFN-α, -β -γ, -λ, -ω), chemokines (MCP-1, IP-10, MIP-1α and β) and immunoglobulins [10, 11, 23, 88–90]. CpG ODNs also stimulates maturation, differentiation, and proliferation of multiple immune cells, including B lymphocytes, monocytes, macrophages and DCs [10, 88–91] and protects these cells from apoptosis [11]. This, in turn, stimulates T cells to secrete additional cytokines and NK cells to secrete IFN-γ and have increased lytic function.

As TLR9 molecules expressed by different species have diverged over evolutionary periods [10] the precise sequence motif (CpG dinucleotide plus flanking sequences) optimal for stimulating immune cells varies between species (Table 1). Cell populations expressing TLR9 also differ between species. In mice, immune cells of the myeloid lineage (monocytes, macrophages, myeloid DCs) express TLR9 whereas, in humans, memory B (but not naïve B cells) [92] and pDCs express TLR9 [12, 82, 88, 89, 91, 93]. The activity of other immune cell subsets such as monocytes, NK cells, γδ T cells, and memory CD8 T cells is increased by CpG ODN via pDC-derived cytokines, but due to the lack of TLR9 expression in these cell subsets, there is no direct effect of CpG ODN on these cells [10, 93, 94]. Human peripheral blood mononuclear cells (PBMC) induce IFN-α and IFN-γ in response to CpG ODN whereas mouse splenocytes induce IFN-γ, IL-12p40 and IL-6. This partial disparity in cytokine induction is due to the mouse splenocyte response being dominated by monocytes (a non-responder cell in humans) [82]. CpG ODNs also upregulate cytokine and chemokines expression in cells of the central nervous system, promote angiogenesis and have an effect on bone formation [89, 95].

CpG ODNs have been synthesized using either a phosphodiester, phosphorothioate or a mixed DNA backbone with the phosphorothioate backbone rendering the CpG ODN more resistant to nucleases [96], extending the plasma half-life from about 5 min to 35–50 h, although a biphasic plasma elimination profile was observed [97], and lowering the dosage required for activity [91]. Liver and kidney have the highest uptake of phosphorothioate-containing CpG ODNs in mice and rats, with a significant amount being detected in the spleen [97]. CpG ODNs accumulate intracellularly with little nuclear uptake [96] except in HeLa cells where K-type CpG ODNs (Table 2) were observed to localize within the nucleus and mitochondria [98]. Dramatically different profiles and kinetics of immune activation have been observed with the various CpG ODN backbones resulting in the classification of three distinct families of CpG ODNs: D-type (CpG-A), K-type (CpG-B) and C-type (CpG-C) (Table 2).

Studies of K-type CpG ODNs indicate that the sequence, number and location of CpG motifs influence the magnitude of the resultant response with CpG motifs at the 5′ end triggering significantly greater immune activation. Addition of extra CpG motifs into D- or C-type CpG ODNs did not improve their activity, likely due to disruption of the palindromic sequence [88, 99]. K-type CpG ODNs may preferentially trigger early type I IFN production, whereas D-type CpG ODNs may be able to support late type I IFN production via the IFN-αβ-mediated feedback loop [93].

### 3.1. CpG ODN as prophylaxis

**Figure 2.**
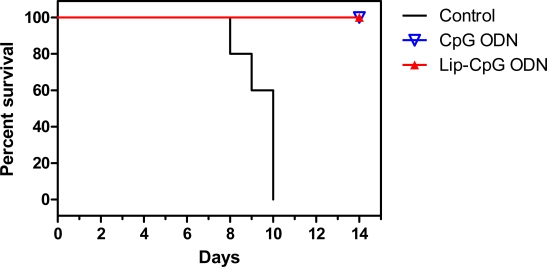
Efficacy of CpG ODN prophylaxis against influenza viral challenge. Mice were given 5 μg (0.25 mg/kg body weight) of free or liposome-encapsulated CpG ODN i.n. 4 d prior to infection with influenza A/PR/8/34. Liposomes were composed of DMTAP/CH/DOPC (molar ratio of 25:50:25). Mice (n=5) were monitored daily for appearance, weight and survival. Graph shows the percentage of mice surviving 14 days after challenge [103]. The experiment was done twice.

Currently CpG ODN-induced activation of innate immunity is being investigated in a wide range of models for protection against a variety of pathogens and for therapeutic activity against cancer and allergy [12, 90]. As the CpG ODN-stimulated immunomodulatory cascade peaks at 2–3 d and persists for several weeks, it has been suggested that CpG ODNs must be administered early (preferably before infection) to protect against rapidly lethal pathogens whereas treatment of slowly growing pathogens can be delayed until several weeks after challenge [89]. In a study evaluating the efficacy of CpG ODN against influenza, mice given 5 μg of a K-type CpG ODN 4 d prior to infection with a lethal dose of influenza A/PR/8/34 (H1N1) survived the viral challenge whereas PBS-treated mice did not (Figure 2), thus demonstrating that CpG ODN pre-treatment is efficacious against influenza viral infection [103].

CpG ODNs appear to enhance the immune response even when the response is impaired due to age or disease. CpG ODN treatment of splenocytes from senescence-accelerated SAM-P1 strain of mice increased IFN-γ and administration *in vivo* generated virus-specific cytotoxic T lymphocyte responses, NK cell activation, virus-specific Ig isotype switch from IgG1 to IgG2a, increased viral clearance and survival following influenza viral challenge.

This suggests that CpG ODNs could contribute to the development of a protective strategy in immunocompromised elderly persons [104]. Although SIV-infected macaques have no detectable IFN-γ production, treatment with D-type CpG ODNs increased IFN-α and reduced *Leishmania amazonensis*-induced lesions when treated at -3 and +3 d, whereas those treated with K-type CpG ODNs had no reduction in infection although IL-6 and cell proliferation was similar to healthy macaques [100]. Caution must be taken in extrapolating animal studies to humans because PBMC from healthy and HIV-infected donors had similar *in vitro* responses to K- and D-type CpG ODNs although the magnitude of the response to D-type CpG ODNs was reduced in HIV-infected donors relative to healthy donors [100]. A clinical study on patients with various types of B cell non-Hodgkin’s lymphoma found that most B cell malignancies (with the exception of plasmacytoma) responded to CpG ODNs by up-regulating expression of co-stimulatory and antigen-presenting molecules, by increasing expression of CD20 and by proliferation, although the proliferative response was less than in normal B cells [105]. These results suggest that it may be possible to use CpG ODNs as adjuvants to expand the potency and efficacy of antiviral vaccines in the population that is most at risk and yet has the least effective immune response to vaccination.

CpG ODNs, when administered prior to pathogen challenge, have been shown to provide complete protection against *Listeria monocytogenes* [101, 106–109], *Francisella tularensis* [107] and herpes simplex virus 2 (HSV-2) [9] in mice, *Escherichia coli* [110] in chickens and *Leishmania major* [100, 111] in Rhesus macaques. When the CpG ODN was administered following infection, complete protection was observed in mice challenged with *Leishmania major* [112]. Complete protection was also observed against *Mycobacterium tuberculosis* [113] however, the CpG ODN had to be given prior to and following infection. CpG ODNs provided partial protection to mice infected with *Plasmodium yoelii* [114, 115], Friend virus [116], HSV-2 [117], Ebola virus [114] or RML scrapie prion [118], to chickens infected with *Eimeria* coccidiosis [119] or to Rhesus macaques infected with *Leishmania amazonensis* [101]. The disparate results observed with mice infected with HSV-2 can likely be attributed to the timing of CpG ODN administration with treatment 24 h prior to infection resulting in complete protection and treatment 2–6 h post-infection resulting in partial protection, although the strain of mouse used could also bias the results with BALB/c and Swiss Webster mice being used, respectively.

### 3.2. CpG ODN as an adjuvant

CpG ODNs increase influenza-specific antibody production when co-administered with inactivated influenza virus, influenza protein or plasmid-based vaccines. Intranasal delivery of formalin-inactivated influenza virus vaccine/CpG ODN mixture enhanced production of influenza–specific antibodies in the serum, saliva and genital tract with seven-fold higher antibody levels detected in mice administered vaccine with CpG ODN [120, 121]. Intranasal delivery of plasmid DNA encoding influenza A/PR/8/34 hemagglutinin (HA) administered with or without K-type CpG ODN afforded protection against a lethal dose of influenza A/PR/8/34 with survivors having elevated anti-HA IgG2a titres. Anti-HA IgG2b titres were also increased but only in CpG ODN treated mice [103]. Covalent linkage of CpG ODN to influenza virus HA resulted in secretion of high levels of IFN- and HA-specific IgG2a antibodies [122] suggesting that crosslinking CpG ODNs to peptides may be an efficient adjuvant method. Encapsulation of CpG ODN and influenza subunit vaccine within liposomes further enhances vaccine potency while reducing the number of administrations. Three to twelve weeks post-vaccination, mice treated with liposome-encapsulated vaccine plus CpG ODN had up to 30 times higher serum and mucosal IgG2a and IgA levels [123].

In a double-blind study, 1 mg of CpG ODNs were co-administered with a commercial trivalent killed split influenza vaccine (Fluarix, SmithKline Beecham). Inclusion of CpG ODNs did not increase the antibody response of naïve recipients when compared to Fluarix alone but did significantly increase anti-HA titers among subjects with pre-existing anti-influenza antibodies. PBMCs from CpG ODN-vaccinated subjects responded to *in vitro* re-stimulation by secreting significantly higher levels of IFN-γ than PBMCs from control vaccinees [124]. Vaccinees given CpG ODN plus either a full or a reduced (0.1) dose of Fluarix had similar hemagglutinin inhibition and anti-HA antibody titres, but antigen-specific IFN-γ secretion from PMBC was decreased in the reduced Fluarix dose group [125]. This suggests that CpG ODN adjuvants can potentially expand the number of people that can be immunized when vaccine supply is limiting.

Human studies using CpG ODN 1018 ISS (immunostimulatory DNA sequence), a 22-mer K-type CpG ODN (Dynavax Technologies) [126, 127] or CPG 7909 (ProMune, Coley Pharmaceuticals) [102, 128–130] have been conducted. CpG ODN co-administered with alum-absorbed HBsAg increased the pool of high-avidity antibodies in an antigen and isotype-specific manner [128] and co-administration with a commercial hepatitis vaccine (Energix B, GlaxoSmith Kline) generated protective levels of anti-HBs antibodies within two weeks of the priming vaccine, with subjects receiving higher doses of CpG ODN having higher rates of positive cytotoxic T cell lymphocyte responses [131]. In phase I clinical studies, seroprotective anti-HBsAg antibody titers were observed after a single dose of rHBsAg plus 3 mg 1018 ISS in 87.5% of subjects whereas those not receiving 1018 ISS did not produce protective antibodies [126]. CpG ODN 1018 ISS has also been evaluated in phase I clinical studies as an adjuvant to the anti-CD20 chimeric monoclonal antibody rituximab (Genetech, Inc.) in patients with relapsed non-Hodgkins lymphoma [127].

In addition to influenza and hepatitis B, CpG ODNs have also been evaluated for their adjuvant potential with protein vaccines targeting hepatitis C virus [117], herpes simplex virus -1 (HSV-1) [132], SARS-CoV [133, 134], bovine herpesvirus [135], simian immunodeficiency virus [136], *Plasmodium yoelii* [137] and melanoma antigen [129] and antibody therapy [127, 138]. In contrast to mice, protocols for immunizing humans and livestock require higher doses of CpG ODN to exert adjuvant activity [132].

### 3.3 CpG ODN safety

Most adverse events associated with CpG ODN co-administration with various vaccines were predominantly short-lived reactions (i.e. pain and erythema) at the injection site and flu-like symptoms [90, 102, 126, 127, 131, 139]. However, concerns relating to CpG ODN-induced production of IL-6 and blockade of apoptotic death of activated lymphocytes, functions that predispose to the development of autoimmune disease by facilitating the persistence of self-reactive lymphocytes, have been raised [89, 90]. Some studies have found that repeated injection of immunostimulatory doses of CpG DNA does not appear to induce or accelerate systemic autoimmune disease [140–142], however, allergic encephalomyelitis [143, 144], autoimmune myocarditis [145], joint inflammation [146] and overproduction of TNF-α which can cause life-threatening toxic shock [90, 147–149] have been demonstrated. Immunosuppression was observed in mice given high (60 μg) doses of CpG ODN daily [150]. Since a single 5 μg dose of CpG ODN was able to afford protection against influenza [103] it would be interesting to know whether daily treatment with a low dose of CpG ODN also induced immunosuppression.

As with poly (ICLC), liposome-encapsulation of CpG ODNs has been used to reduce toxicity and prolong exposure due to gradual release of the CpG ODN from liposomes [132, 151]. Mice treated with liposome-encapsulated CpG ODN 4 d prior to infection with a lethal dose of influenza A/PR/8/34 survived the influenza viral challenge with a lower weight loss and faster recovery than mice treated with free CpG ODN, suggesting that liposome-encapsulation decreases CpG ODN toxicity [103]. CpG ODN encapsulated within stabilized antisense-lipid particles increased plasma concentrations of IL-6, -12, IFN-γ, monocyte chemoattractant protein-1 and TNF-α to a greater extent than unencapsulated CpG ODN, with encapsulated phosphodiester CpG ODNs strongly stimulating cytokine induction at the early time points [152]. This suggests that liposome-encapsulation can be used to decrease dosage required for activity.

Encapsulation of CpG ODNs within large (1.5 μm) multilamellar liposomes can change the type of response observed depending on what is co-administered. In mice, co-administration with either a subunit influenza vaccine or HBsAg demonstrated a Th1-dominant or a mixed Th1/Th2 response in the influenza and hepatitis B models, respectively [123]. In cells lacking TLR9, such as prostate cancer PC3 cells, treatment with phosphorothioate CpG ODNs had no effect unless co-administered with Lipofection transfection agent suggesting that phosphorothioate CpG ODNs could also function in a TLR9-independent manner and that liposome-encapsulation of CpG ODNs could potentially expand the variety of cell types responding to the CpG ODN [153]. Co-delivery of CpG ODN adjuvants and antigens in nanospheres has also been shown to be an efficient approach for immunization [154].

Conjugation of ODNs or ligands to CpG ODNs has been used in attempts to improve efficacy. In mice, a CpG ODN-antigen conjugate inhibited influenza virus more efficiently than the co-administration of CpG ODN and antigen [155]. The location of ligand conjugation is important as CpG ODNs have reduced immunostimulatory activity when compounds were conjugated 5′-5′, although conjugation of small molecules (i.e. phosphorothioate groups) had an insignificant effect. Conjugation of an ODN or a ligand through the 3′ end of CpG ODN (3′-3′ linkage) has no effect on immunostimulatory activity [156]. Two CpG ODNs linked 3′-3′ are termed immunomers. A synthetic nucleoside “R” with a bicyclic heterobase [1-(2′-deoxy-β-_D_-ribofuranosyl)-2-oxo-7-deaza-8-methyl-purine] has been used to replace the C in the CpG dinucleotide motif and has been evaluated in both a RpG ODN and an immunomer. RpG ODNs activated NF-kÂ and mitogen-activated protein kinase pathways and RpG immunomers induced high levels of IL-12 and IFN-γ in a time- and concentration-dependent fashion in mouse splenocytes costimulated with IL-2. Significantly, immunomers containing GTRGTT and GARGTT were recognized to a similar extent by both mouse and human immune systems, stimulated proliferation of PBMCs and prevented conalbumin- and ragweed allergen-induced allergic inflammation in mice [157].

## 4. TLR7 agonists

TLR7 is an intracellular TLR, located on endosomal membranes, which recognizes ssRNA in a sequence-independent manner as long as the RNA contains several uridine residues in close proximity to each other [158, 159]. Nucleosides and nucleotides from intracellular pathogens, guanine nucleoside analogues, stabilized immunoregulatory RNA, short dsRNA oligonucleotides containing several uridine residues in close proximity (i.e. short interfering RNA (siRNA)) [160] and imidoazoquinoline-based compounds such as 852A and imiquimod (IMQ) [161] are also recognized by TLR7. As with TLR9, pDC are the primary IFN-α producing cells following exposure to a TLR7 agonist and stimulation of TLR7 results in induction of IL-6, IL-12, MIP-1α, MIP-1β, TNF-α and IFN-β among others [161–165]. Single-stranded RNA induction of TLR7 also stimulates autophagy, a cell-autonomous innate defence mechanism for elimination of intracellular pathogens, in macrophages. This induction of autophagy appears to be effective in eliminating intracellular microbes, even when the target pathogen is normally not associated with TLR7 signalling [166].

TLR7 is essential for influenza viral recognition and inflammatory cytokine production by murine neutrophils [159, 167]. Mice pretreated with a TLR7 agonist (4-[6-amino-8-hydroxy-2-(2-methoxy-ethoxy)purin-9-ylmethyl]benzaldehyde) – mouse serum albumin conjugate prior to influenza A H1N1 viral challenge had a significant delay in mortality relative to those not receiving the conjugate [168] suggesting that stimulation of the influenza-binding receptor before infection can improve the outcome Intranasal administration of the synthetic TLR7/8 agonist 3M-011 significantly inhibited H3N2 influenza viral replication in the nasal cavity of rats when administered between 72 h before and 6 h after viral challenge. Viral inhibition correlated with the ability of the TLR7/8 agonist to stimulate type I IFN and other cytokines such as TNF-α, IL-12, and IFN-γ from rat PBMC. The activity of the TLR7/8 agonist resulted in greater inhibition of viral titers compared to rat recombinant IFN-α administered in a comparable dosing regimen [169].

There are, however, some serious concerns relating to stimulation of TLR7 receptors. *In vivo* studies in systemic lupus erythematosus (SLE) mouse models demonstrate an essential role for TLR7 in the generation of RNA-containing antinuclear antibodies and deposition of pathogenic immune complexes in the kidney, with TLR7 recognizing RNA- and DNA-containing autoimmune complexes and TLR9 amplifying the autoimmune response [170]. Transgenic analysis of TLR7 determined that a modest increase in TLR7 expression resulted in spontaneous development of autoimmunity and a substantial increase in TLR7 expression caused fatal acute inflammation and profound DC dysregulation, indicating that TLR7 must be tightly regulated in order to prevent spontaneous triggering of harmful autoreactive and inflammatory responses [171]. Inhibition of TLR7 and TLR9 with the immunoregulatory sequence 954 inhibited the induction of IFN-α by human pDC in response to DNA and RNA viruses and immune complexes from SLE patients and reduced SLE disease severity [172]. In humans, PBMCs isolated from females produced significantly higher IFN-α levels after TLR7 stimulation than did PBMCs isolated from males although there was no difference in TNF-α production between cells isolated from both sexes. This sex-dependent activation of IFN-α by TLR7 may explain the higher prevalence of SLE in females and the reported decrease in therapeutic efficacy of synthetic TLR7 ligands in males [173].

## 5. Influenza A H5N1: cytokine response

Influenza A is a highly contagious single-stranded RNA virus that infects both the upper and lower respiratory tracts of humans. During a single-cycle infection, human viruses preferentially infect nonciliated cells, whereas avian viruses primarily infect ciliated cells. This correlates with the predominant localization of receptors for human (α-2,6-linked sialic acids) and avian (α-2,3-linked sialic acids) viruses on nonciliated and ciliated cells, respectively [174]. Sialic acid linked to galactose via α-2,3 glycosidic bonds, is a cellular receptor located in the eye, which may account for the ocular tropism exhibited by zoonotic avian influenza A viruses such as H5N1 in Hong Kong in 1997, N7N2 in the U.S.A. in 2003, H7N7 in the Netherlands in 2003 and H7N3 in Canada in 2004 [175]. Infiltration of lymphocytes, neutrophils, and macrophages in the lungs and production of reactive oxygen species, which contributes to pulmonary tissue damage, is observed [176, 177].

Influenza virus infection induces expression of a variety of factors including IL-1β, -6, -18, TNF-α, Fas ligand, IFN regulatory factor (IRF)-1, IFN-α, -β, eotaxin (eosinophil chemoattractant), RANTES, TGF-β, dsRNA dependent protein kinase (PKR), indolamine 2,3-deoxygenase (IDO) and 2′-5′-oligoadenylate synthetase (2–5 OAS) [178–181]. Inflammatory-mediated apoptosis is also induced [180–182]. IL-2, produced by T cells specific for influenza A virus, is involved in T cell dependent IFN-γ production by NK cells, suggesting that at an early stage of recurrent viral infection, NK cell-mediated innate immunity to the virus is enhanced by pre-existing virus specific T cells [183]. A recent study showed that chickens infected with influenza H9N2 viral strains were resistant to H5N1 influenza viral challenge. This cross-protection was mediated by T cells bearing CD8(+) and T-cell receptor (TCR) α/β Vβ1 subset [184]. Protective immunity was closely related to the percentage of CD8(+) T cells expressing IFN-γ in the lung, rather than in the spleen, suggesting that pulmonary cellular immunity may be very important in protecting naïve natural hosts against lethal influenza viruses [184].

There are significant differences in cytokine responses between H5N1 (A/HK/483/97, A/Vietnam/1194/04 and A/Vietnam/3046/04) and H1N1 influenza viruses, primarily in relation to IFN-β, IL-6, IP-10 and RANTES which are elevated post-infection in human type II pneumocytes, but significantly more so in H5N1 infected cells, with more recent H5N1 viruses from Vietnam (H5N1/04) being more potent at inducing IP-10 [18]. A comparative study using quantitative PCR and cDNA arrays demonstrated that H5N1/97 viruses induced much higher gene transcription of proinflammatory cytokines, particularly TNF-α and IFN-β, than did H3N2 or H1N1 influenza viruses in human primary monocyte-derived macrophages *in vitro* [185]. Inactivation of the virus by UV irradiation prior to infection of alveolar epithelial cells abolished cytokine induction suggesting that virus replication was required for cytokine induction [18]. Transforming growth factor β (TGF-β), a potent proinflammatory cytokine that activates monocytes to induce the expression and release of various growth factors and inflammatory mediators, is induced by infection with non-HK-origin H5N1 avian influenza viruses as early as 8-h p.i. and continues to increase for at least 96 h. In contrast, HK-origin influenza virus infected mice showed no increase in TGF-β activity suggesting that HK viruses fail to activate latent TGF-β [186].

Influenza H5N1 infection in birds is systemic, characterized by hemorrhage and edema resulting from virus replication in the endothelium [187]. Systemic infection has also been observed in cynomolgus macaques and humans [188, 189]. Vascular endothelial growth factor (VEGF), produced primarily but not exclusively in alveolar epithelial cells, is induced in a time-and dose-dependent manner by IFN-γ, IL-1β and TNF-α, increasing microvascular permeability and contributing to pulmonary edema [190]. In mice infected with influenza H5N1/97, virus-infected cells initially appeared in the respiratory tract and later could be detected in neurons, glial and ependymal cells of the central nervous system [191].

H5N1 influenza viruses can be further classified into high and low pathogenicity viruses. The difference in host response to the lethal and nonlethal H5N1 influenza virus is likely due to the non-structural (NS) protein [185, 192, 193]. Mice infected with a lethal (A/Hong Kong/483/97) H5N1 influenza virus had a significant decrease in the total number of circulating leukocytes (primarily lymphocytes) as early as 2 d p.i. and a reduction in the number of CD4(+) and CD8(+) T cells [194]. IL-1β, IFN-γ and MIP-1α in lung and lymphoid tissue were elevated in mice infected with either a lethal (A/Hong Kong/483/97) or nonlethal (A/Hong Kong/486/97) H5N1 influenza virus although the degree of elevation was lower in mice infected with the lethal strain [195–197]; whereas, TNF-α and MIP-2 levels were elevated in a similar manner by both strains [196, 197]. It has been suggested that TNF-α may contribute to early disease severity whereas IL-1 may play a role in viral clearance late in H5N1 infection [195]. Mice infected with the lethal H5N1 influenza virus HK483 also had increased concentrations of IL-1β, TNF-α, IFN-γ, MIP-1α and MIP-2 in the brain, and apoptosis in the spleen and lung [194]. Although A/HK/483/97 infected mice showed no evidence of virus-induced encephalitis, the local synthesis of TNF-α or IL-1 within the brain could contribute to anorexia, weight loss and death [198]. The lethal H5N1 influenza virus appears to possess the capacity to limit the induction of immune responses by targeting and destroying lymphocytes resulting in aberrant production of cytokines in serum and tissues (“cytokine storm”). In addition to the cytokine storm, systemic viral dissemination and alveolar flooding due to inhibition of cellular sodium channels contribute to the lethality of influenza H5N1 disease [18, 185, 189, 199–201].

## 6. Influenza A H5N1: use of non-specific immune stimulators

In humans, influenza H5N1 virus mediates a cytokine storm characterized by insensitivity to the antiviral effects of IFN (possibly due to insufficient production) and increased concentration of IFN-β, IP-10, RANTES, IL-6 and TNF-α in the lung and macrophages. In mice, decreased CD4(+) and CD8(+) T cells, apoptosis of lymphocytes in the spleen and lung and detection of cytokines in the brain are associated with influenza H5N1 pathology. Theoretically, for non-specific immune stimulators to be effective in influenza H5N1 viral infection, they should overcome the insensitivity to IFN and should prevent apoptosis of lymphocytes without contributing further to cytokine dysregulation. Prevention of apoptosis of T cells could potentially increase TNF-β, IFN-γ, IL-2, -3, -4, -5, -9, -10 and -15. Preferably, TNF-α would not be induced by the non-specific immune stimulator as it is already highly induced by influenza A H5N1 viral infection, although mice deficient for TNF-α or its receptors had no reduction in mortality when infected with A/Vietnam/1203/04 (H5N1) suggesting that TNF-α alone is not responsible for the increased mortality of H5N1 influenza viruses [201]. Inhibition of the cytokine response by corticosterone, the natural mouse glucocorticoid, was not sufficient to prevent death, regardless of when the drug was administered, [201] suggesting that factors other than the cytokine storm are important for lethality.

**Figure 3.**
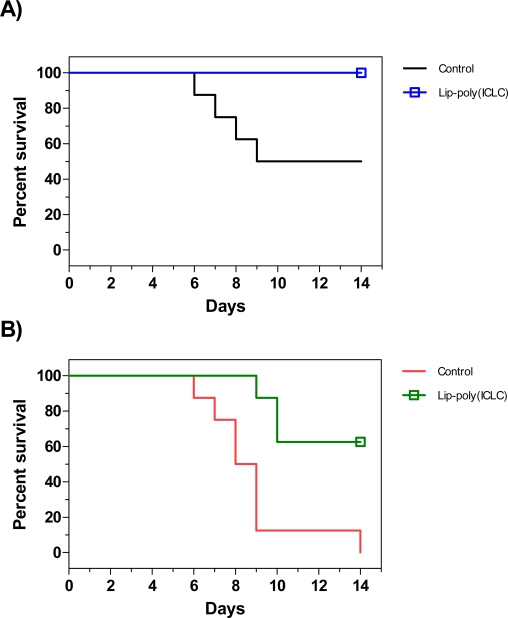
Efficacy of liposome-encapsulated poly (ICLC) prophylaxis against influenza H5N1 viral challenge. Mice were given 20 μg (1 mg/kg body weight) of liposome-encapsulated poly (ICLC) intranasally (i.n.) 48 and 8 h prior to challenge with a 1 LD50 (A) or 4 LD50 (B) of influenza A/H5N1/chicken/Henan. Liposomes were composed of PC/CH/SA in a 7:2:1 molar ratio. Mice (n=8) were monitored daily for appearance, weight and survival. Graph shows the percentage of mice surviving 14 days after challenge [215]. The experiment was done twice.

TLR7 activation by influenza virus stimulates cytokine production [159, 167], whereas a MyD88-dependent pathway distinct from the TLR7 pathway appears to be involved in B cell responses to influenza virus [202, 203]. The potent IFN-α induction by C- and D-type CpG ODNs in human pDCs and B cells is markedly reduced by stimulation of TLR7, without affecting IL-6 secretion or B cell proliferation [204–206]. This suggests that a negative feedback mechanism has evolved which could act to prevent levels of IFN-α secretion that are detrimental to the host. Experimental analysis of sequential activation of TLR7, 8 and 9 in HEK293 cells demonstrated that activation of TLR9 inhibited TLR7 activation but not vice versa [207]. This suggests that CpG ODNs could be prophylactic agents against H5N1 influenza viral infection. TLR3 agonist plus TLR7/8 agonists, in the presence of the membrane permeability enhancer DOTAP, had an additive effect on IFN-α/β responses in human PBMCs [204]. Treatment of mice with liposome-encapsulated poly (ICLC) has been evaluated for efficacy against influenza A/H5N1/chicken/Henan (Figure 3). Mice given one LD50 of the virus had a 50% survival rate whereas those given two doses of liposome-encapsulated poly (ICLC) had a 100% survival rate. When the virus dose was increased to 4 LD50, survival of treated mice decreased to 63% whereas the untreated mice succumbed to infection. This demonstrates that non-specific immune stimulators may be an effective prophylaxis against a pandemic strain of influenza.

Similar to influenza H5N1 virus, respiratory syncytial virus (RSV) is also a poor inducer of IFN-α/β and is partially resistant to IFNs antiviral activity. When poly (ICLC), an IFN-α inducer, was given before RSV infection, mice had a milder disease and/or faster recovery with increased IFN production and reduced viral replication [208]. However, when either poly (ICLC) or CpG ODN was administered 48 h post-RSV infection, IFN-α production was almost completely inhibited [208]. In human pDC, the TLR9-dependent IFN-inducing pathways were abolished by infection with measles virus and RSV A2 with the RSV-mediated effects being attributed to the NS protein of RSV through interference with activation of the essential IFN transcription factor IRF-3 [209]. It has not yet been demonstrated whether influenza H5N1 affects TLR3/TLR9 signalling pathways.

Should an influenza H5N1 pandemic occur, all age groups would be affected. Influenza traditionally disproportionately affects the young and the elderly, with both the innate and adaptive immune systems being affected by aging. In the innate immune system, the functions of NK cells, macrophages (fewer number and less efficient antigen presentation) and neutrophils (impaired chemotaxis, degranulation and phagocytosis) are decreased with aging [210]. Age-related changes in the adaptive immune system include diminished/altered cytokine patterns (Th2 bias), reduction in clonal expansion and function of antigen-specific T and B cells and a decline in antigen-presenting cell function [210]. Humoral immunity also exhibits changes albeit to a lesser extent, particularly the diminished ability to generate high-affinity protective antibodies against infectious agents. Splenic and activated peritoneal macrophages from aged mice express significantly lower levels of all TLRs and macrophages from aged mice secrete significantly lower levels of IL-6 and TNF-α when stimulated with known TLR ligands [210]. Poly (ICLC) was able to effectively protect aged mice against lethal murine cytomegalovirus infection and effectively induced IFN [211]. In immature and aging mice, treatment with CpG hastened maturation of DCs and recovery/enhancement of the Th1 type response, respectively [212–214]. Similarly, immune compromised SCID mice were almost completely protected against murine cytomegalovirus infection by poly (ICLC) and poly (ICLC) was able to induce IFN and NK cell cytotoxicity in these mice [211]. These results suggest that, although TLR3 and TLR9 receptors are reduced during the aging process, poly (ICLC) and CpG ODN still have potential to be effective.

## 6. Conclusions

In light of current concerns regarding a potential influenza pandemic, both poly (ICLC) and CpG ODNs appear to have potential to stimulate the innate immune system and thus provide protection. As poly (ICLC) stimulates IL-12 and CpG ODN stimulates IL-6, -12 and TNF-α, they would theoretically be effective adjuvants for generating cross-protective antibodies. In the advent of a pandemic vaccine being produced, poly (ICLC) and CpG ODNs have adjuvant potential thus expanding the number of people that could be immunized. Use of poly (ICLC) and CpG ODNs as adjuvants requires lower doses than when used solely for prophylaxis, thus alleviating some of the toxicity concerns associated with their use.

Poly (IC) and CpG ODN co-administration demonstrate synergy in nitric oxide, IL-12, TNF-α and IL-6 production in murine macrophages and *in vivo*, with synergism being mediated by the paracrine/autocrine effects of IFN-β [216]. Bovine macrophages and DCs also show a synergistic response with regard to TNF production when poly (IC) and CpG ODN are co-utilized [217]. Synergy of poly (ICLC) and CpG ODN with respect to protection from influenza viral challenge, either alone or as an adjuvant, remains to be evaluated.

## Figures and Tables

**Table 1.** Optimal CpG Sequence Motifs.

Species	Motif	Reference
Rodents	5′ PuPuCGPyPy 3′	[82, 83]
Canines	5′ ATCGAT 3′	[84]
Primates	5′ GTCGTT 3′	[82, 85, 86]

**Table 2.** Features of the three families of CpG ODNs.

Type	Features	Comments
D-type (CpG- A)	- mixed phosphodiester / phosphorothioate backbone - 1–2 CpG dinucleotides in a central phosphodiester region flanked on both the 5′ and 3′ ends with phosphorothioate nucleotides - CpG motif is located within a palindromic sequence - run of G’s at 3′ end [88, 93]	- stimulate NK cells to produce IFN-γ [13, 88, 93] - stimulate pDCs to produce large amounts of IFN-α, IFN-β and TNF-α [13, 88, 93, 94] - stimulate IFN-α and IFN-γ secretion and maturation of human DCs *in vitro* [100] - induce IFN-α within the first 12 h with considerable amounts still produced at 24–48 h [93] - indirectly activate monocytes to differentiate into myeloid DCs and produce chemokines (IL-10) [88] - trigger the maturation of APCs [88] - do not stimulate B-cells or other subsets of DCs [89] - active in mice [89], nonhuman primates [100] - best activity in humans [13, 88, 93]
K-type (CpG- B)	- phosphorothioate backbone - multiple TCGT/A motifs[88, 89]	- stimulate strong B-cell and NK cell activation [13] - activate pDCs to produce IFN-α, -β, IL-6, -8, TNF-α and IP-10 but very little IFN-γ [89, 93, 101] - IFN-α induction is relatively low and limited to the first 12 h [93] - short-lived induction of IFN-γ, IL-6 and TNF-α in BALB/c mice but IL-12 remains elevated for at least 8 d [101] - stimulate B-cells to secrete IL-6, IL-10 and IgM [88, 89, 102] - stimulate cytokine production [13] - induce cell proliferation and IL-6 production from human PBMCs [100] - active in mice [89], human PBMC *in vitro* [100] - poorly active in primates [89]
C-type (CpG- C)	- phosphorothioate backbone - palindromic sequence - no poly(G) stretch - TCGTCG at the 5′ end - frequently contains a K-type motif (GTCGTT) [13, 88]	- stimulate strong B-cell and NK cell activation [13] - stimulate pDCs to produce IFN-α [13, 88] - potent Th1 adjuvant [13] - stimulate B-cells to secrete IL-6, IL-10 and IgM [88, 89, 102]
